# Phasing and imputation of single nucleotide polymorphism data of missing parents of biparental plant populations

**DOI:** 10.1002/csc2.20409

**Published:** 2021-06-14

**Authors:** Serap Gonen, Valentin Wimmer, R. Chris Gaynor, Ed Byrne, Gregor Gorjanc, John M. Hickey

**Affiliations:** ^1^ The Roslin Institute and Royal (Dick) School of Veterinary Studies University of Edinburgh Easter Bush Research Centre Midlothian EH25 9RG UK; ^2^ KWS SAAT SE Grimsehlstr. 31 Einbeck 37574 Germany; ^3^ KWS‐UK Ltd 56 Church Street Thriplow Hertfordshire SG8 7RE UK

## Abstract

This paper presents an extension to a heuristic method for phasing and imputation of genotypes of descendants in biparental populations so that it can phase and impute genotypes of parents that are ungenotyped or partially genotyped. The imputed genotypes of the parent are used to impute low‐density (Ld) genotyped descendants to high density (Hd). The extension was implemented as part of the AlphaPlantImpute software and works in three steps. First, it identifies whether a parent has no or Ld genotypes and identifies its relatives that have Hd genotypes. Second, using the Hd genotypes of relatives, it determines whether the parent is homozygous or heterozygous for a given locus. Third, it phases heterozygous positions of the parent by matching haplotypes to its relatives. We measured the accuracy (correlation between true and imputed genotypes) of imputing parent genotypes in simulated biparental populations from different scenarios. We tested the imputation accuracy of the missing parent's descendants using the true genotype of the parent and compared this with using the imputed genotypes of the parent. Across all scenarios, the imputation accuracy of a parent was >0.98 and did not drop below ∼0.96. The imputation accuracy of a parent was always higher when it was inbred than outbred. Including ancestors of the parent at Hd, increasing the number of crosses and the number of Hd descendants increased the imputation accuracy. The high imputation accuracy achieved for the parent translated to little or no impact on the imputation accuracy of its descendants.

## Core Ideas

- Hd SNP data in plant breeding populations is increasingly valuable for genomic selection.
- Hd genotyping thousands of candidates is expensive, imputation is a cost‐effective alternative.
- This paper presents an extension to a heuristic method for phasing and imputation of genotypes.
- Across all scenarios, imputation accuracy of a parent was >0.98 and did not drop below ∼0.96.
- High imputation accuracy for the parent translated to no impact on offspring imputation.

**Abbreviations**
Hd: high density
Ld: low density
SNP: single nucleotide polymorphism.

## 1 INTRODUCTION

This paper presents an extension to a heuristic method for phasing and imputation of genotypes of descendants in biparental populations. The original method assumes that parents of the descendants to be imputed are phased and genotyped at high density (Hd). The extension presented herein removes this assumption by phasing and imputing genotypes of parents of biparental populations that are fully ungenotyped or partially genotyped. The imputed genotypes of the parent are then used to impute low‐density (Ld) genotyped descendants of the biparental population to Hd.

High‐density single nucleotide polymorphism (SNP) data in plant breeding populations is increasingly valuable for genomic selection and for identifying regions of the genome that underlie traits of interest in genome‐wide association studies (Bernardo & Yu, 2007; Hamblin, Buckler, & Jannink, 2011). One of the major barriers to the adoption of genomic selection in plant breeding programs is that the number of selection candidates that would need to be genotyped at Hd in each cycle can be very large (Heffner, Lorenz, Jannink, & Sorrells, 2010).

In livestock and human populations, an effective strategy to overcome this cost barrier is to genotype a subset of the population at Hd and to use this data for imputation of the rest of the population genotyped at Ld. The adoption of this strategy has been enabled by the development of imputation tools that leverage pedigree relationships or population‐level linkage information for fast and accurate genotype imputation (Antolín, Nettelblad, Gorjanc, Money, & Hickey, 2017; Cleveland & Hickey, 2013; Hickey et al., 2011; Kong et al., 2008; Sargolzaei, Chesnais, & Schenkel, 2011; VanRaden, Sun, & O'Connell, 2015).

In most plant breeding populations, a small number of selected parents are crossed to generate large numbers of biparental populations. Therefore, Hd genotyping of all parents and Ld genotyping of focal individuals (i.e., descendants that are the imputation targets) could be an effective low‐cost strategy in these populations (Gorjanc et al., 2017b; Jacobson, Lian, Zhong, & Bernardo, 2014, 2015). Plant breeding programs have three main features that make them more amenable to imputation that livestock. These are (a) fully or almost fully inbred parents, (b) small numbers of meiosis separating parents and descendants who are to have genotypes imputed, and (c) different crossing structures (e.g., selfing, double haploids). To our knowledge, very few imputation tools designed to leverage these features to enable fast and accurate genotype imputation have been developed. We recently presented a fast, computationally efficient, and accurate heuristic genotype imputation method implemented in AlphaPlantImpute (Gonen et al., 2018) that explicitly leverages these features of plant breeding programs to maximize the accuracy of imputation. Using simulated data, we showed that an average accuracy of imputation of 0.96 could be achieved for a scenario where F_2_ individuals to be imputed were genotyped with 50 markers per chromosome and both parents were inbred and genotyped at 25,000 markers per chromosome.

The drawback of our previous algorithm is the requirement that both parents of each biparental population are known and have phased genotypes available at Hd. Although this is normally the case when parents are inbred, pedigree errors, sample loss, or mislabeling or poor DNA quality can mean that one or both parents may have fully or partially missing genotype data. Additionally, if genotyping resources are limiting, breeders may choose not to genotype a parent that has only been used to in one or two crosses. Furthermore, even if parents have Hd genotypes available, unless they are fully inbred (i.e., homozygous at every locus and therefore all genotypes are phased de facto) it is unlikely that they have phased genotypes available for use in imputation.

**Core Ideas**

- Hd SNP data in plant breeding populations is increasingly valuable for genomic selection.
- Hd genotyping thousands of candidates is expensive, imputation is a cost‐effective alternative.
- This paper presents an extension to a heuristic method for phasing and imputation of genotypes.
- Across all scenarios, imputation accuracy of a parent was >0.98 and did not drop below ∼0.96.
- High imputation accuracy for the parent translated to no impact on offspring imputation.

This paper presents an extension to our previous algorithm in AlphaPlantImpute for phasing and imputation of Hd genotypes of parents of biparental populations that are missing or that only have Ld genotypes available. The extension requires that some relatives of the parent (e.g., descendants, ancestors, siblings) have Hd genotypes. The extension has three steps. First, it identifies whether a parent has no or Ld genotypes available and all of its relatives that have Hd genotypes. Second, using the Hd information of relatives, it determines whether the parent is homozygous or heterozygous for a given locus. Third, it phases heterozygous positions of the parent by matching haplotypes to its relatives.

We tested the accuracy of imputing missing parent genotypes using the extension to AlphaPlantImpute in simulated biparental populations from different scenarios. These scenarios varied in the levels of inbreeding in the missing parent, whether the parent had no genotypic data or was genotyped at Ld, the number of crosses that the parent was used and whether the ancestors of the parent had Hd genotypes available. We calculated the accuracy of imputation of the missing parent within each scenario as the correlation between the true and imputed genotypes. We also tested the accuracy of imputation of the missing parent's descendants using the true genotype of the parent compared with using the imputed genotypes of the parent. Our results show that across all scenarios, the accuracy of imputation of a parent was consistently high. The imputation accuracy of a parent was always higher when it was inbred than when it was outbred and when it had Ld genotypes than when it was not genotyped. Including ancestors of the parent at Hd, increasing the number of crosses and increasing the number of Hd descendants all increased the accuracy of imputation. The high imputation accuracy achieved for the parent across all scenarios had little or no impact on the accuracy of imputation of its descendants at Ld, which remained high.

## 2 MATERIALS AND METHODS

### 2.1 Definitions

A focal individual is a descendant individual that is to be imputed. Parent A is the missing parent that is the target of imputation. In our test datasets, 25,000 SNP markers is considered Hd and is the target for imputation, and 50 SNP markers is considered as Ld genotypes in the text below are coded as 0, 1, and 2, where 0 and 2 represent homozygotes for the two alternative alleles, and 1 represents heterozygotes.

### 2.2 Description of the method

The original imputation method in AlphaPlantImpute assumed that parents of focal individuals to be imputed have Hd phased and imputed genotypes available. The original method first phases and assigns parent of origin to the haplotypes of focal individual at Ld using allele linkage information between two neighbouring SNPs. The parent‐of‐origin information at Ld for each haplotype is used as anchor points to impute each focal individual to Hd. The imputation method in AlphaPlantImpute did not consider the case where parents do not have Hd phased and imputed genotypes available.

We present an extension to the original imputation method in AlphaPlantImpute to phase and impute parents of biparental populations that are missing or that have Ld genotypes available. The algorithm begins from the base generation of the pedigree and performs checks to ensure that they have Hd phased and imputed genotypes before moving on to their offspring. First, AlphaPlantImpute identifies parents with missing genotypes or unphased genotypes (hereafter described for a single parent referred to as Parent A). Second, AlphaPlantImpute gathers Hd genotype information of all known relatives of or mated (i.e., crossed) to Parent A. Relatives include ancestors, siblings, and descendants. AlphaPlantImpute then uses any genotype information available on Parent A and its relatives to first impute missing genotypes and then phase heterozygous genotypes of Parent A.

#### 2.2.1 Parent A not genotyped

In livestock, the next generation are produced by a single cross of two ancestors. This means that SNPs where both ancestors are homozygous for the same genotype (i.e., both are genotype 0 or genotype 2) and where ancestors are opposing homozygotes (i.e., one is genotype 0 and the other is 2) can be confidently imputed in their offspring. In plant breeding populations, individuals are often the product of a single cross to produce F_1_ individuals followed by many rounds of selfing. This means that if an offspring (in this case Parent A) has no genotypes but has ancestors genotyped at Hd, the only SNPs that can be confidently imputed are those where both of its ancestors are homozygous for the same allele. These SNPs are phased de‐facto.

If Parent A has Hd descendants and mates, this information is used to phase and impute genotypes for Parent A in the following three steps:

1. SNPs where Parent A is likely to be homozygous based on allele frequencies in descendants are inferred. For example, if all Hd descendants are fixed for the 0 allele, then Parent A is likely to be genotype 0.
2. SNPs where Parent A is likely to be heterozygous based on genotype frequency distortion in descendants are inferred. This is calculated using a chi‐square test of observed genotype counts to expected genotype counts given observed allele frequencies. If there is significant distortion and the mate is homozygous, then Parent A is heterozygous. If there is no significant distortion and the mate is homozygous (i.e., 0 or 2) then Parent A is imputed to the opposing homozygous genotype (i.e., 2 or 0).
3. Genotypes of all Hd descendants and mates at SNPs where Parent A is imputed as heterozygote are collated. These SNPs are used as anchor points in the heuristic imputation algorithm of AlphaPlantImpute (Gonen et al., 2018) to determine parent of origin for the haplotypes of all descendants at Hd. These haplotypes are used to derive consensus haplotypes for Parent A.

#### 2.2.2 Parent A has Ld genotypes

If Parent A has Ld genotypes and has ancestors genotyped at Hd, AlphaPlantImpute uses the Ld genotypes in the heuristic imputation algorithm as described in Gonen et al. (2018). Briefly, the Ld genotypes serve as anchor points for defining parent of origin for the haplotypes of Parent A and simultaneously phasing and imputing it to Hd.

If Parent A has Hd descendants and mates, the genotypes of Parent A are imputed using the following four steps:

1. SNPs at which Parent A is genotyped at Ld are identified.
2. The genotypes of descendants and mates at these Ld SNPs are used as anchor points in the existing heuristic imputation algorithm of AlphaPlantImpute (Gonen et al., 2018) to determine parent of origin for the haplotypes of all descendants. Briefly, SNPs at Ld, where the two parents are homozygous, are used to assign parent of origin to the haplotypes of the descendants. For example, if Parent A has genotype 0, Parent B has genotype 2, and the descendant has genotype 2, both haplotypes of that descendant are assigned to Parent B. Heterozygous genotypes of the descendant are phased using linkage information and parent‐of‐origin assignment of neighbouring loci.
3. The haplotypes of descendants at Hd assigned to Parent A are used to derive consensus haplotypes for Parent A.
4. The genotypes of Parent A are filled as the sum of the two derived haplotypes.

If Parent A has Hd ancestors, descendants and mates then a consensus of the phased and imputed genotypes using only ancestor information or using only descendant information is derived. Where they disagree, the haplotype and genotype are set to missing.

### 2.3 Examples of implementation: Description of datasets

To test the imputation accuracy of this modification of AlphaPlantImpute, testing datasets of biparental populations from different scenarios were simulated. These scenarios varied in the levels of inbreeding in the missing parent, whether the parent had no genotypes or was genotyped at Ld, the number of crosses that the parent was used in and whether the ancestors of the parent had Hd genotypes available. A description of the general structure and simulation method of the different scenarios is given below.

### 2.4 Simulation of genomic data

Sequence data for 100 base haplotypes for a single chromosome were simulated using the Markovian Coalescent Simulator (Chen, Marjoram, & Wall, 2009) and AlphaSimR (Faux et al., 2016). The base haplotypes were 10^8^ base pairs in length, with a per site mutation rate of 1.0 × 10^−8^ and a per site recombination rate of 1.0 × 10^−8^, resulting in a chromosome size of 1 Morgan (M). The effective population size (*N*_e_) was set at specific points during the simulation to mimic changes in *N*_e_ in a crop such as maize (*Zea mays L*.). These set points were 100 in the base generation, 1000 at 100 generations ago, and 10,000 at 2,000 generations ago with linear changes in between. The resulting whole‐chromosome haplotypes had ∼80,000 segregating sites in total.

### 2.5 Simulation of a pedigree

The base generation of 1,000 inbred individuals was initiated from the resulting whole‐chromosome haplotypes generated by the Markovian Coalescent Simulator and AlphaSimR (Gaynor, Gorjanc, Wilson, Money, & Hickey, 2019). Two individuals from this base generation (denoted B and C) were crossed to generate 1,000 F_1_ individuals. These individuals were selfed for *n* rounds and one individual was selected to be Parent A. The number of rounds of selfing (*n*) was 100 if Parent A was simulated to be fully inbred or was 0 if Parent A was simulated to be outbred. Depending on the scenario, Parent A was crossed to 1, 2, 3, or 4 individuals (denoted D, E, F, G) from the initial base generation to generate 1,000 of F_1_ individuals. The F_1_ individuals were selfed to generate 1,000 F_2_ individuals. These were the descendants used for imputation of Parent A.

In the base generation, individuals had their chromosomes sampled from the 100 base haplotypes. In subsequent generations the chromosomes of each individual were sampled from parental chromosomes with recombination, resulting in a chromosome size of 1 Morgan (M). Recombinations occurred with a 1% probability per cM and were uniformly distributed along the chromosome.

### 2.6 Simulated SNP markers

A total of 25,000 SNP markers at Hd and 50 SNP markers at Ld for the single chromosome was simulated. The SNP markers were selected as a set of markers that segregated in the parents and that were evenly distributed across the chromosome. The Ld SNP markers were selected as a subset of the Hd SNP markers. Allele frequencies were allowed to vary between 0.01 and 0.50.

### 2.7 Scenarios

The imputation accuracy of Parent A was assessed in eight different scenarios. Scenarios were designed to test the effect of including or excluding ancestors of Parent A (hereafter referred to as Grandparent 1 and Grandparent 2) and the effect of having genotype information of F_2_ individuals from one, two, three, or four crosses of Parent A with Parents B, C, D, and E. From each cross, 10 F_2_ individuals were selected as Hd descendants. The remaining 990 were F_2_ focal individuals genotyped at Ld. In all scenarios, Parent A could be either inbred or outbred and could be either genotyped at Ld or not genotyped. One hundred replications of each scenario were performed, and the average and standard deviation of each replication is reported in the results.

Scenarios 1, 2, 3, and 4 excluded Grandparent 1 and Grandparent 2. Scenarios 5, 6, 7, and 8 included Grandparent 1 and Grandparent 2. Scenarios 1 and 5 had information from one cross (Parent A × Parent B). Scenarios 2 and 6 had information from two crosses (Parent A × Parent B and Parent A × Parent C). Scenarios 3 and 7 had information from three crosses (Parent A × Parent B, Parent A × Parent C, and Parent A × Parent D). Scenarios 4 and 8 had information from four crosses (Parent A × Parent B, Parent A × Parent C, Parent A × Parent D, and Parent A × Parent E).

In addition to the imputation accuracy of Parent A, the accuracy of imputing the 990 F_2_ focal individuals genotyped at Ld to Hd using the phased and imputed genotypes of Parent A was assessed. This was compared with the imputation accuracy that would have been achieved if genotypes of Parent A were known and not imputed.

### 2.8 Analysis

Imputation of Parent A was performed using information across all crosses from descendants genotyped at Hd and information from Parents B, C, and D and Grandparents 1 and 2, if available. Imputation of the 990 F_2_ focal individuals genotyped at Ld was performed within a cross using the heuristic imputation method of AlphaPlantImpute described in Gonen et al., 2018. The imputation accuracy was calculated as the correlation between the true and imputed genotypes. The imputation yield was calculated as the number of SNPs with imputed genotypes divided by the total number of SNPs at Hd (i.e., 25000). In all scenarios, Grandparents 1 and 2 and Parents B, C, D, and E were assumed genotyped at Hd.

## 3 RESULTS

Unless otherwise stated, all results presented below had 10 Hd descendants per cross.

### 3.1 Effect of whether Parent A is inbred or outbred

**FIGURE 1:**
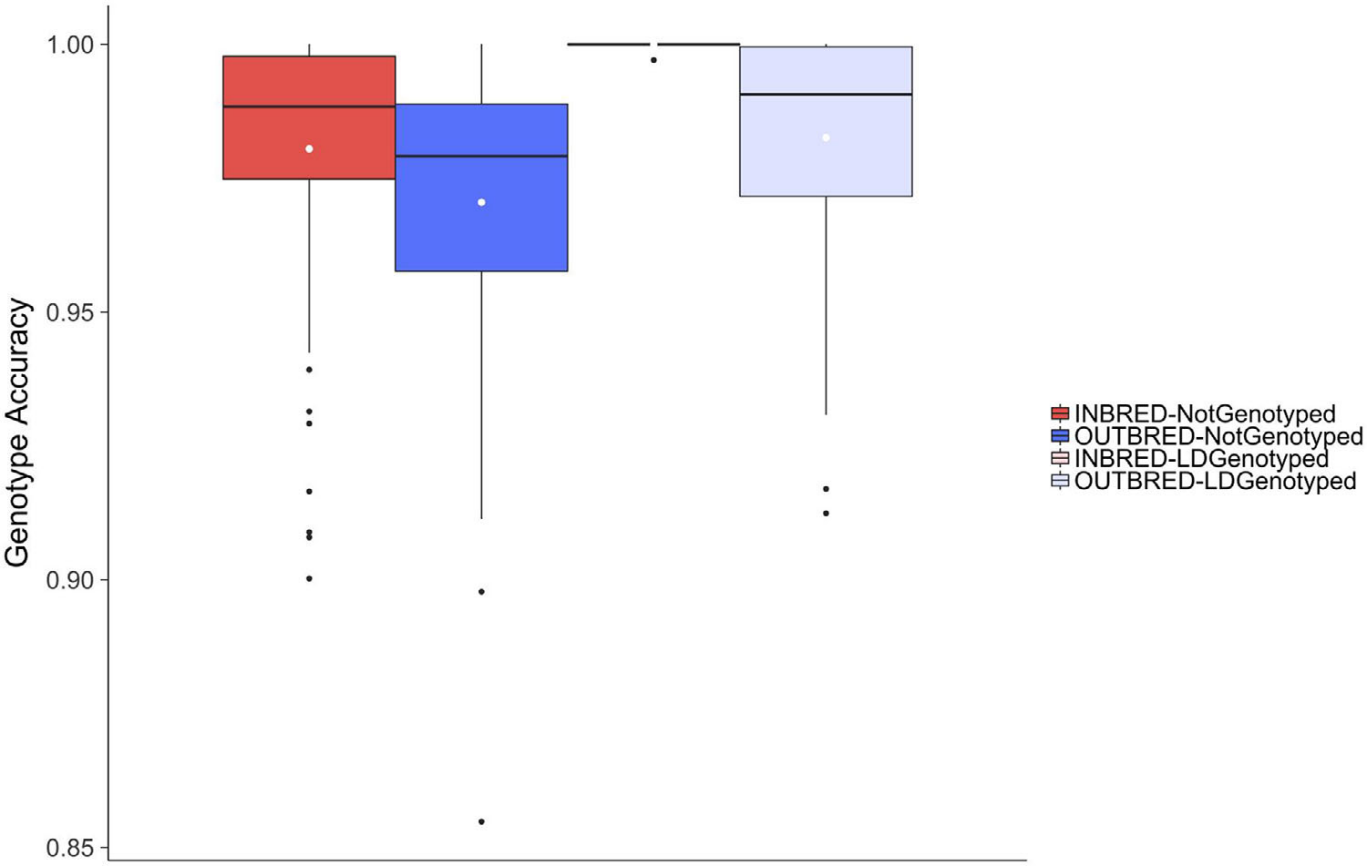
Effect of whether Parent A is inbred or outbred and whether Parent A has no or low‐density (Ld) genotypes

The imputation accuracy of Parent A was always higher when it was inbred than when it was outbred but the differences were small. Figure 1 plots the genotype accuracy for Parent A in Scenario 1. Figure 1 shows that when Parent A had no genotypes, the accuracy of imputation was slightly (1.01 times) higher when it was inbred than when it was outbred (0.980 ± 0.023 vs. 0.970 ± 0.026). When Parent A had Ld genotypes, the accuracy of imputation was slightly (1.02 times) higher when it was inbred than when it was outbred (0.999 ± 0.0003 vs. 0.983 ± 0.020). For all cases, the yield of imputation was 100%.

### 3.2 Effect of whether Parent A has Ld genotypes or not

The imputation accuracy of Parent A was always higher when it had Ld genotypes than when it had no genotypes, but the differences were small. Figure 1 shows that when Parent A was inbred, the accuracy of imputation was slightly (1.02 times) higher when it had Ld genotypes than when it had no genotypes (0.999 ± 0.0003 vs. 0.980 ± 0.023). When Parent A was outbred, the accuracy of imputation was slightly (1.01 times) higher when it had Ld genotypes than when it had no genotypes but the differences were small (0.983 ± 0.020 vs. 0.970 ± 0.026).

### 3.3 Effect of including Grandparent 1 and Grandparent 2 at Hd

**FIGURE 2:**
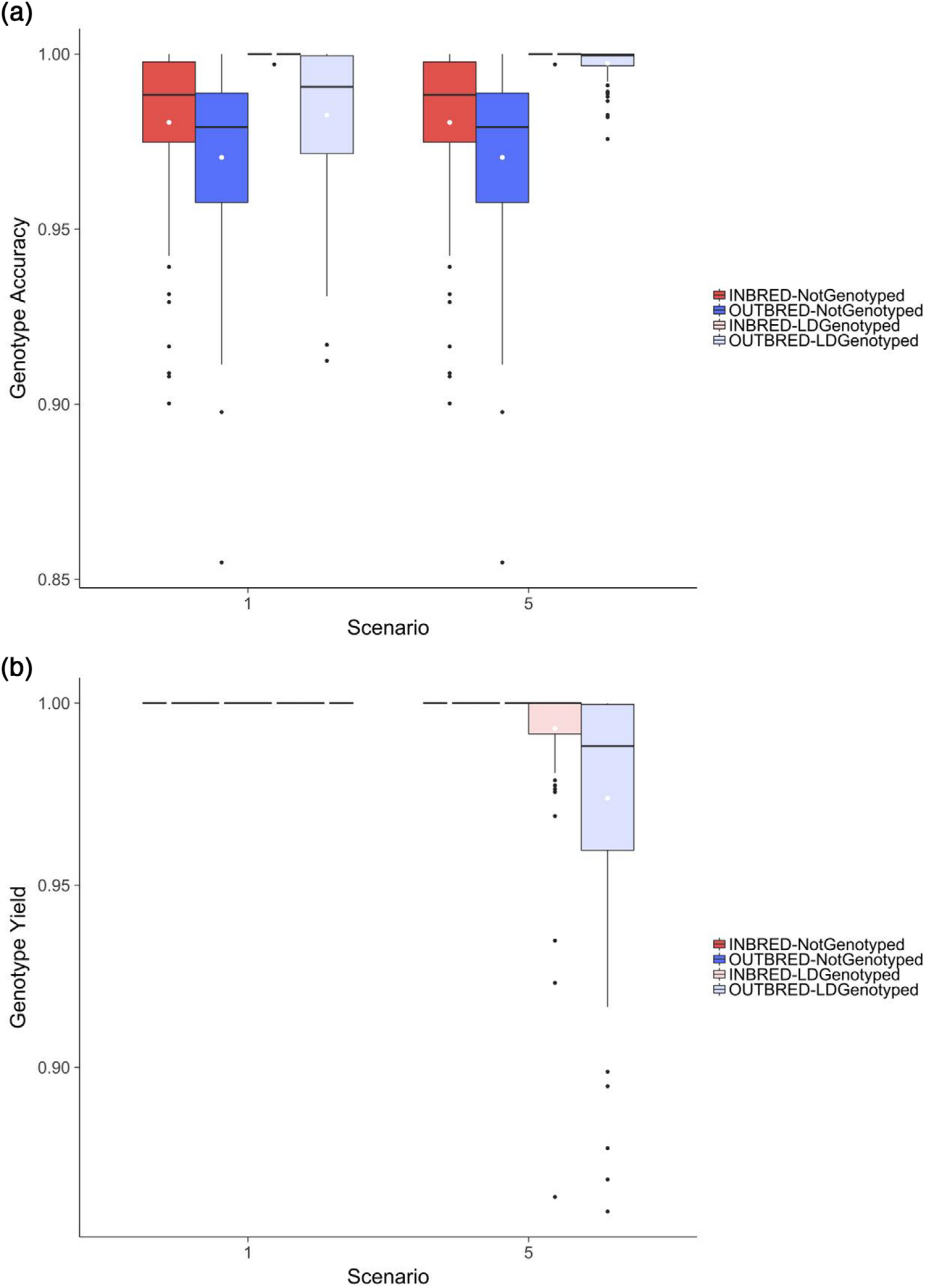
Effect of including ancestors of Parent A at high density

Including Grandparent 1 and Grandparent 2 increased the accuracy of imputation when Parent A has some Ld genotypes, but the differences were small. When Parent A had no genotypes, the accuracy of imputation was the same regardless of whether Grandparent 1 and Grandparent 2 were included or excluded. Figure 2 is similar to Figure 1 and plots the genotype accuracy (Figure 2a) and genotype yield (Figure 2b) for Parent A in Scenarios 1 and 5. Figure 2a shows that the main benefit of including Grandparent 1 and Grandparent 2 for increasing the imputation accuracy was when Parent A was outbred and had Ld genotypes. In this case, the accuracy of imputation of Parent A was slightly (1.02 times) higher when Grandparent 1 and Grandparent 2 were included than when they were excluded (0.983 ± 0.020 vs. 0.997 ± 0.004). However, this increase in accuracy was at the expense of yield. Figure 2b shows that when Parent A was outbred and had Ld genotypes, the yield was 100% when Grandparent 1 and Grandparent 2 were excluded and was 97.4% when Grandparent 1 and Grandparent 2 were included.

### 3.4 Effect of the number of crosses with Parent A

**FIGURE 3:**
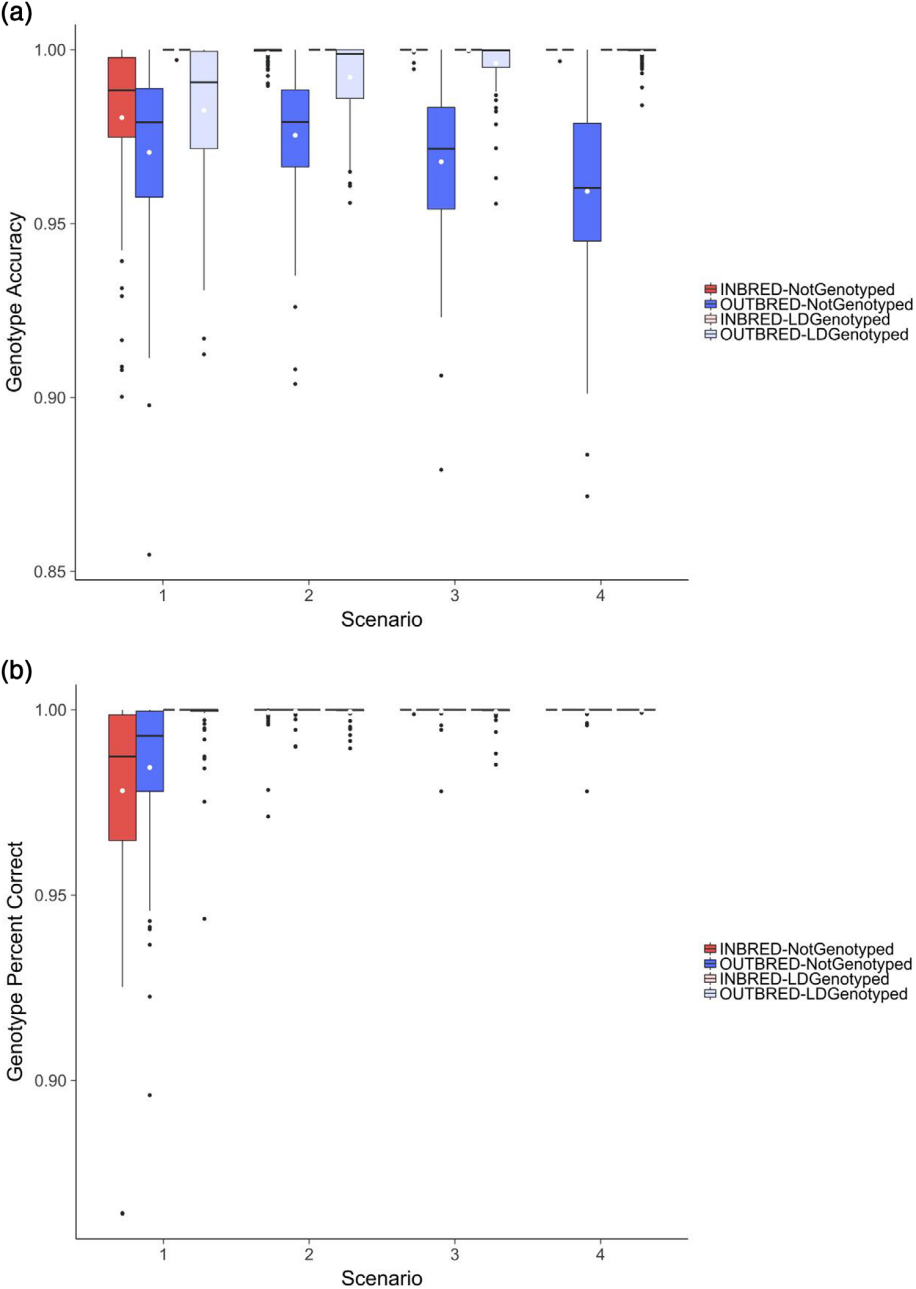
Effect of the number of crosses and number of high‐density descendants per cross

Increasing the number of crosses that Parent A was used in increased the accuracy of imputation but the differences were small. Figure 3a is similar to Figure 1 and plots the genotype accuracy for Parent A in Scenarios 1, 2, 3, and 4. Figure 3a shows that increasing the number of crosses from one in Scenario 1 to two in Scenario 2 increased the imputation accuracy regardless of whether Parent A was inbred or outbred, or had no genotypes or had Ld genotypes. When Parent A was inbred, the accuracy of imputation was slightly (1.02 times) higher in Scenario 2 than in Scenario 1 when it had no genotypes (0.980 ± 0.023 vs. 0.999 ± 0.002) and was just slightly higher when it had Ld genotypes (0.999 ± 0.0003 vs. 1.0 ± 0.00). When Parent A was outbred, the accuracy of imputation was 1.01 times higher in Scenario 2 than in Scenario 1 when it had no genotypes (0.970 ± 0.026 vs. 0.975 ± 0.019) and was slightly (1.01 times) higher when it had Ld genotypes (0.983 ± 0.020 vs. 0.992 ± 0.011). For all cases, the yield of imputation was 100%.

Increasing the number of crosses that Parent A was used in increased the accuracy of imputation most when Parent A was outbred and had Ld genotypes, but the differences were small. Figure 3a shows that when the number of crosses increased from one in Scenario 1 to four in Scenario 4, the accuracy of imputation was slightly (1.02 times) higher in Scenario 4 than in Scenario 1 when Parent A was outbred and had Ld genotypes (0.983 ± 0.020 vs. 0.999 ± 0.002).

Figure 3a also shows that increasing the number of crosses that Parent A was used in decreased the accuracy of imputation when Parent A was outbred and had no genotypes but the differences were small. When the number of crosses increased from one in Scenario 1 to four in Scenario 4, the accuracy of imputation was slightly (1.01 times) higher in Scenario 1 than in Scenario 4 (0.970 ± 0.026 vs. 0.959 ± 0.026).

### 3.5 Effect of number of descendants with Hd genotypes

Increasing the number of descendants with Hd genotypes increased the accuracy of imputation of Parent A, but the differences were small. Figure 3b is similar to Figure 3a and plots the genotype accuracy for Parent A in Scenarios 1, 2, 3, and 4 when the number of descendants with Hd genotypes was 50. For example for Scenario 1, when the number of descendants increased from 10 to 50 the accuracy of imputation was 1.01 times higher when Parent A was inbred and had no genotypes (0.980 ± 0.023 vs. 0.988 ± 0.015), was just slightly higher when Parent A was inbred and had Ld genotypes (0.999 ± 0.0003 vs. 1.00 ± 0.00), was 1.02 times higher when Parent A was outbred and had no genotypes (0.970 ± 0.026 vs. 0.990 ± 0.012), and was 1.02 times higher when Parent A was outbred and had Ld genotypes (0.983 ± 0.020 vs. 0.999 ± 0.005). For all cases, the yield of imputation was 100%. Figure 3b also shows that when the number of descendants with Hd genotypes was 50; increasing the number of crosses to two or more resulted in accuracy of imputation for Parent A of >0.999.

### 3.6 Effect of using imputed genotypes or true genotypes of Parent A to impute F_2_ focal individuals

**FIGURE 4:**
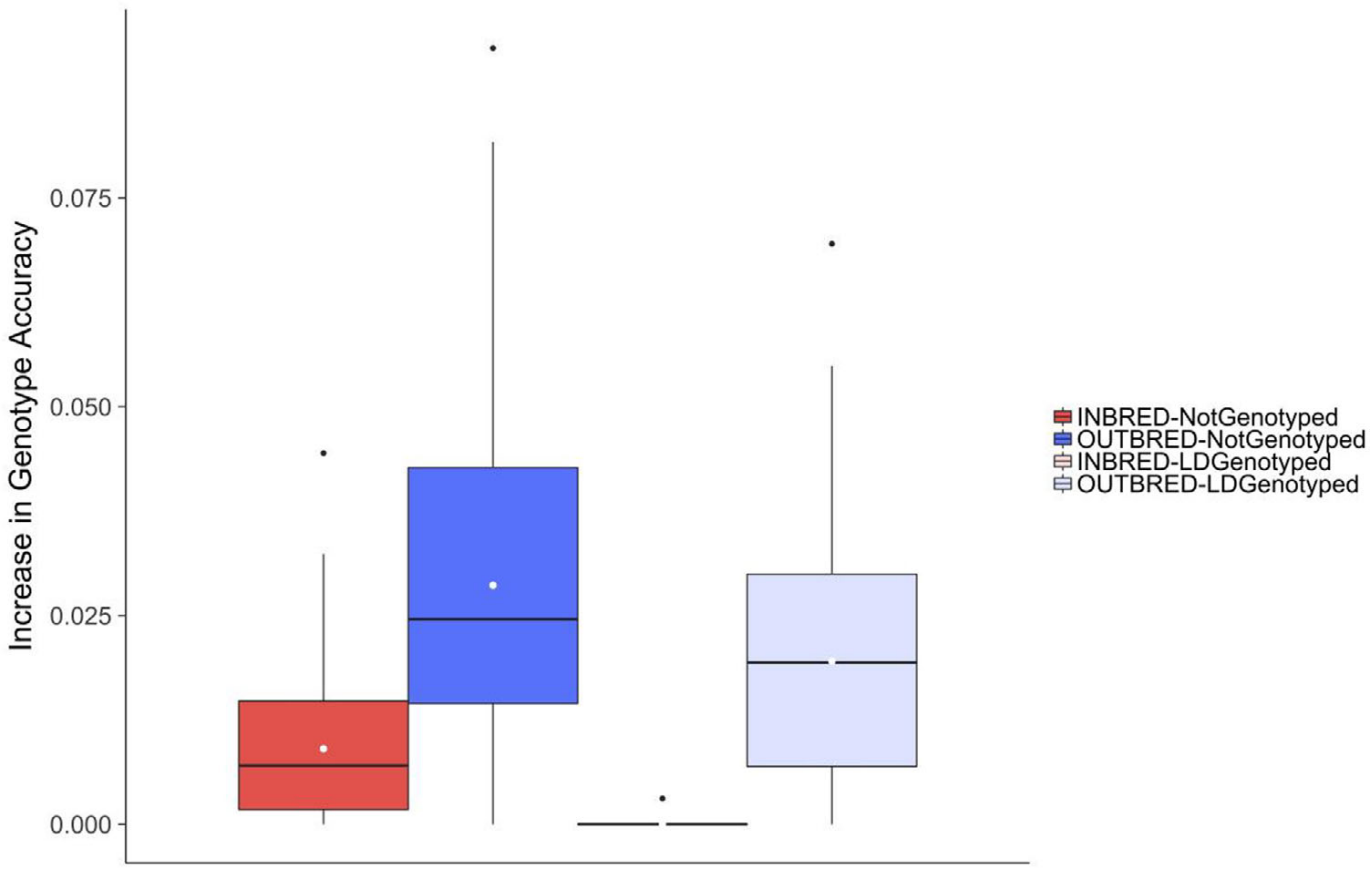
Effect of using imputed genotypes or true genotypes of Parent A to impute F_2_ focal individuals

Using true or imputed genotypes of Parent A had only a small effect on the accuracy of imputation of impute F_2_ focal individuals. Figure 4 plots the increase in imputation accuracy achieved for F_2_ focal individuals for Scenario 1. The increase in imputation accuracy is the difference between the accuracy achieved using true or imputed genotypes for Parent A to impute focal individuals. Figure 4 shows that the increase in imputation accuracy achieved for focal individuals using true genotypes of Parent A compared with using imputed genotypes was minimal regardless of whether Parent A was inbred or outbred or had Ld or no genotypes. The largest increase achieved was when Parent A was outbred and had no genotypes, where an increase of 0.029 was achieved. When Parent A was inbred and had Ld genotypes, there was no increase in the accuracy of imputation of focal individuals when using true or imputed genotypes for Parent A.

## 4 DISCUSSION

Our results highlight two main points for discussion: (a) the performance of AlphaPlantImpute in imputing Parent A and (b) the effect of using imputed genotypes or true genotypes of Parent A to impute F_2_ focal individuals.

### 4.1 Performance of AlphaPlantImpute in imputing Parent A

This paper presents an extension to the original heuristic imputation method in AlphaPlantImpute (Gonen et al., 2018) to phase and impute genotypes for parents of biparental populations that are missing or that have Ld genotypes available. The extension requires that some relatives of the parent (e.g., descendants, ancestors, or siblings) have Hd genotypes. We tested and compared the performance of the algorithm, which we implemented in an updated version of AlphaPlantImpute (Gonen et al., 2018), across a range of scenarios where the parent to be imputed (Parent A) could be inbred or outbred, could have no or Ld genotypes, could be a parent of one or multiple crosses with descendants at Hd, or could have parents with Hd genotypes. In general, across all scenarios, the average accuracy was >0.98 and the average accuracy did not drop below ∼0.96. The yield was 100% for all scenarios apart from when Grandparents 1 and 2 (i.e., the ancestors of Parent A) were included with Hd genotypes. The only scenario where this was not the case was when Grandparents 1 and 2 were included and Parent A was outbred and had Ld genotypes. In this case, the yield dropped to 97%. The reason for this is that this scenario had Hd genotypes available for both Grandparents 1 and 2 and for 10 offspring of Parent A. The heuristic algorithm uses the two sources of information independently to impute Parent A. Where they disagree, even for one haplotype, the genotype is set as missing.

As expected, adding more information from relatives genotyped at Hd increased the accuracy of imputation for Parent A. When Parent A was used in a single cross, including its parents at Hd increased the accuracy of imputation for Parent A, particularly when Parent A was outbred and had Ld genotypes. However, the increase in accuracy when Parent A had Ld genotypes was at the expense of yield. The reason for this decrease in yield is likely caused by disagreement between Parent A genotypes imputed using its descendants genotyped at Hd and genotypes imputed using its parents genotyped at Hd. When Parent A had no genotypes, including its parents at Hd had no effect. This is because the only SNPs that could be filled with confidence were SNPs where its parents were fixed for the same allele.

Increasing the number of crosses that Parent A was used in increased the accuracy of imputation for Parent A when it was inbred or outbred and had Ld genotypes. This likely was due to two reasons. First, the extra Hd information from other crosses increased the ability to call heterozygous SNPs. For example, by chance, within a single cross, one of the haplotypes of Parent A may have been underrepresented or not represented in the descendants selected for Hd genotyping but may have been represented in Hd descendants in the second cross. Second, the Ld genotypes of Parent A were used to assign parent of origin to the haplotypes of Hd descendants. The SNPs that were not informative of parent of origin within one cross may have been informative in another cross, providing extra information on the haplotypes of Parent A. Increasing the number of crosses that Parent A was used in had only a small benefit when Parent A was inbred and had no genotypes. In this case, the accuracy of imputation for Parent A was already ∼0.98 with a single cross and increasing to number of crosses increased the accuracy of imputation for Parent A to >0.999. The only exception to the benefit of increasing the number of crosses was when Parent A was outbred and had Ld genotypes. This could have been caused by incorrect assignment or the inability to assign parent of origin to the haplotypes of Hd descendants, which would result in incorrect or uncalled genotypes for Parent A.

Increasing the number of descendants at Hd within a cross increased the accuracy of imputation across all scenarios. This is expected, since more Hd relatives provides more information for confidently calling the genotypes of Parent A.

Overall, the results suggest that high imputation accuracy of >0.98 and an imputation yield of 100% in almost all cases can be achieved for Parent A by collating Hd genotypes of as many relatives as possible. This is critical for ensuring accurate imputation of descendants genotyped at Ld.

### 4.2 Effect of using imputed genotypes or true genotypes of Parent A to impute F_2_ focal individuals

Using true or imputed genotypes of Parent A had only a small effect on the accuracy of imputation of impute F_2_ focal individuals. The largest increase in imputation accuracy when using true genotypes rather than imputed genotypes for Parent A was observed when Parent A was outbred and not genotyped, but even in this case, the increase was 0.028. The likely reason for the small increase was that the accuracy of imputation of Parent A was, in general, >0.96 across all scenarios. Therefore, our results suggest that some error in the imputation of Parent A is likely to have minimal, if any, effect on the imputation of focal individuals that are its descendants.

### 4.3 Relevance for breeding programs

The use of genomic information in plant breeding populations could have a large impact for informing selection decisions (Bassi, Bentley, Charmet, Ortiz, & Crossa, 2016; Bernardo & Yu, 2007; Daetwyler, Bansal, Bariana, Hayden, & Hayes, 2014; Hamblin et al., 2011; Heffner et al., 2010; Hickey et al., 2014). However, the large cost associated with the large number of candidates that would need to be genotyped in order to leverage the power of genomic selection is still a bottleneck. One way of overcoming this bottleneck would be to genotype the many thousands of selection candidates at Ld and impute them to Hd. To do this, the parents of the candidates need to have phased Hd genotypes available or inferred. Genotyping parents at Hd and inferring phase is theoretically feasible. However, in practice, not all parents will have phased Hd genotypes available because of (a) low quality DNA samples (b) missing DNA samples (for example for older samples), (c) parents that are used in only a single cross may not be worth genotyping, (d) incomplete pedigrees, and (e) pedigree errors. If relatives (e.g., ancestors, offspring, siblings, or mates) of a parent have Hd genotypes available, this information could be used to phase and impute Hd genotypes for the missing parent. The imputed genotypes could then be used to impute any selection candidates that descend from this missing parent. Our simulations show that high imputation accuracy and yield can be obtained for a missing parent, providing a cost‐effective and powerful way of obtaining accurate Hd genotypes for selection candidates that are descendants of the imputed parent.

### 4.4 Software availability

We implemented our method in a software package called AlphaPlantImpute, which is available for download at http://www.AlphaGenes.roslin.ed.ac.uk/AlphaPlantImpute/ along with a user manual.

## 5 CONCLUSIONS

This paper presents an extension to a heuristic method implemented in AlphaPlantImptue so that it can phase and impute genotypes of parents of biparental populations that are fully ungenotyped or partially genotyped. The imputed genotypes of the parent are then used to impute Ld genotyped descendants of the biparental population to Hd. Our results show that the imputation yield was 100% in almost all scenarios. The accuracy of imputation of a parent was >0.98 and did not drop below ∼0.96. The imputation accuracy of a parent was always higher when it was inbred than when it was outbred and when it had Ld genotypes. Including ancestors of the parent at Hd, increasing the number of crosses, and increasing the number of Hd descendants all increased the accuracy of imputation. The high imputation accuracy achieved translated to little or no impact on the accuracy of imputation of its descendants at Ld, which remained high. This extension will be useful in plant breeding populations aiming to incorporate genomic selection for a large number of candidates genotyped at Ld where one of the parents of those candidates has no Hd phased genotypes available.

## AUTHOR CONTRIBUTIONS STATEMENT

SG and JH conceived the method. SG further developed the method, coded the final program, developed the study design and performed the analysis. VW, RCG, EB, and GG contributed to the development of components of the method, to the design and analysis and to the interpretation of the results and provided comments on the manuscript. SG and JH wrote the first draft. All authors read and approved the final manuscript.

## CONFLICT OF INTEREST

The authors declare that they have no conflict of interest.
